# Estimating the Prevalence of Asymptomatic COVID-19 Cases and Their Contribution in Transmission - Using Henan Province, China, as an Example

**DOI:** 10.3389/fmed.2021.591372

**Published:** 2021-06-23

**Authors:** Chunyu Li, Yuchen Zhu, Chang Qi, Lili Liu, Dandan Zhang, Xu Wang, Kaili She, Yan Jia, Tingxuan Liu, Daihai He, Momiao Xiong, Xiujun Li

**Affiliations:** ^1^Department of Biostatistics, School of Public Health, Cheeloo College of Medicine, Shandong University, Jinan, China; ^2^Department of Applied Mathematics, Hong Kong Polytechnic University, Hong Kong, China; ^3^Department of Biostatistics and Data Science, School of Public Health, University of Texas Health Science Center at Houston, Houston, TX, United States

**Keywords:** COVID-19, asymptomatic cases, infectious dynamic model, the effective reproductive number, prevention and control measures

## Abstract

**Background:** Novel coronavirus disease 2019 (COVID-19), caused by the severe acute respiratory syndrome coronavirus 2 (SARS-COV-2), is now sweeping across the world. A substantial proportion of infections only lead to mild symptoms or are asymptomatic, but the proportion and infectivity of asymptomatic infections remains unknown. In this paper, we proposed a model to estimate the proportion and infectivity of asymptomatic cases, using COVID-19 in Henan Province, China, as an example.

**Methods:** We extended the conventional susceptible-exposed-infectious-recovered model by including asymptomatic, unconfirmed symptomatic, and quarantined cases. Based on this model, we used daily reported COVID-19 cases from January 21 to February 26, 2020, in Henan Province to estimate the proportion and infectivity of asymptomatic cases, as well as the change of effective reproductive number, *R*_*t*_.

**Results:** The proportion of asymptomatic cases among COVID-19 infected individuals was 42% and the infectivity was 10% that of symptomatic ones. The basic reproductive number *R*_0_ = 2.73, and *R*_*t*_ dropped below 1 on January 31 under a series of measures.

**Conclusion:** The spread of the COVID-19 epidemic was rapid in the early stage, with a large number of asymptomatic infected individuals having relatively low infectivity. However, it was quickly brought under control with national measures.

## Introduction

In December 2019, cases of pneumonia with an unknown cause were reported. The disease was later named as novel coronavirus disease 2019 (COVID-19), caused by the severe acute respiratory syndrome coronavirus-2 (SARS-COV-2) (1, 2). The rapid increase in confirmed cases and subsequent secondary outbreaks in many countries caused concern on an international scale. As a result, the World Health Organization declared the COVID-19 outbreak a Public Health Emergency of International Concern on January 31, 2020 and eventually classified it as a pandemic on March 11, 2020 (3). As of July 19, 2020, 14 million COVID-19 cases and 597,583 deaths have been confirmed globally, including 85,937 confirmed cases in China (4). Although the number of confirmed cases was staggering, only the sicker part of those infected were being reported. Li et al. used a metapopulation model to estimate that 86% of the infections (presumably of mild symptoms or asymptomatic) before January 23, 2020 were undetected in Wuhan, China (5); Chinazzi et al. used a GLEAM model to estimate that only one out of four cases were confirmed in Mainland China by February 1, 2020 (6, 7). Hao et al. used a SAPHIRE model to estimate that 87% of the infections before March 8, 2020 were unascertained in Wuhan, China (8). And some even suggested that most infections were caused by undetected cases (5, 9). A significant proportion of these undetected infected individuals were asymptomatic (8). In one documented case, a patient who disclaimed all symptoms and showed a normal chest radiography had multiple PCR cycle counts consistent with that of symptomatic patients (10), suggesting such patients are somewhat infectious (11).

The proportion of asymptomatic cases is a critical epidemiological characteristic that modulates the pandemic potential of the emergent respiratory virus, and is an important parameter in estimating the disease burden (5, 12–14). Estimating the proportion of asymptomatic cases will improve the understanding of COVID-19 transmission and spectrum of presentation, thereby providing insight into the spread of epidemics (14). But the estimated proportion of asymptomatic infected individuals varied widely from place to place. A recent analysis of 21 retrieved reports by the Centre for Evidence-Based Medicine in Oxford found that estimates of asymptomatic COVID-19 cases ranged from 5 to 80% (15). Meanwhile, most studies only showed that asymptomatic infected individuals are less contagious than symptomatic ones (16, 17). Only one previous study clearly showed that the asymptomatic cases could be one quarter as infectious as symptomatic cases in Ningbo, China (18). Therefore, it is important to estimate the proportion and infectivity of asymptomatic cases in various regions. Taking Henan Province as an example, we used a model-inference framework to explore the proportion and infectivity of asymptomatic cases, so as to estimate the prevalence of COVID-19.

## Methods

### Study Area

**Figure 1:**
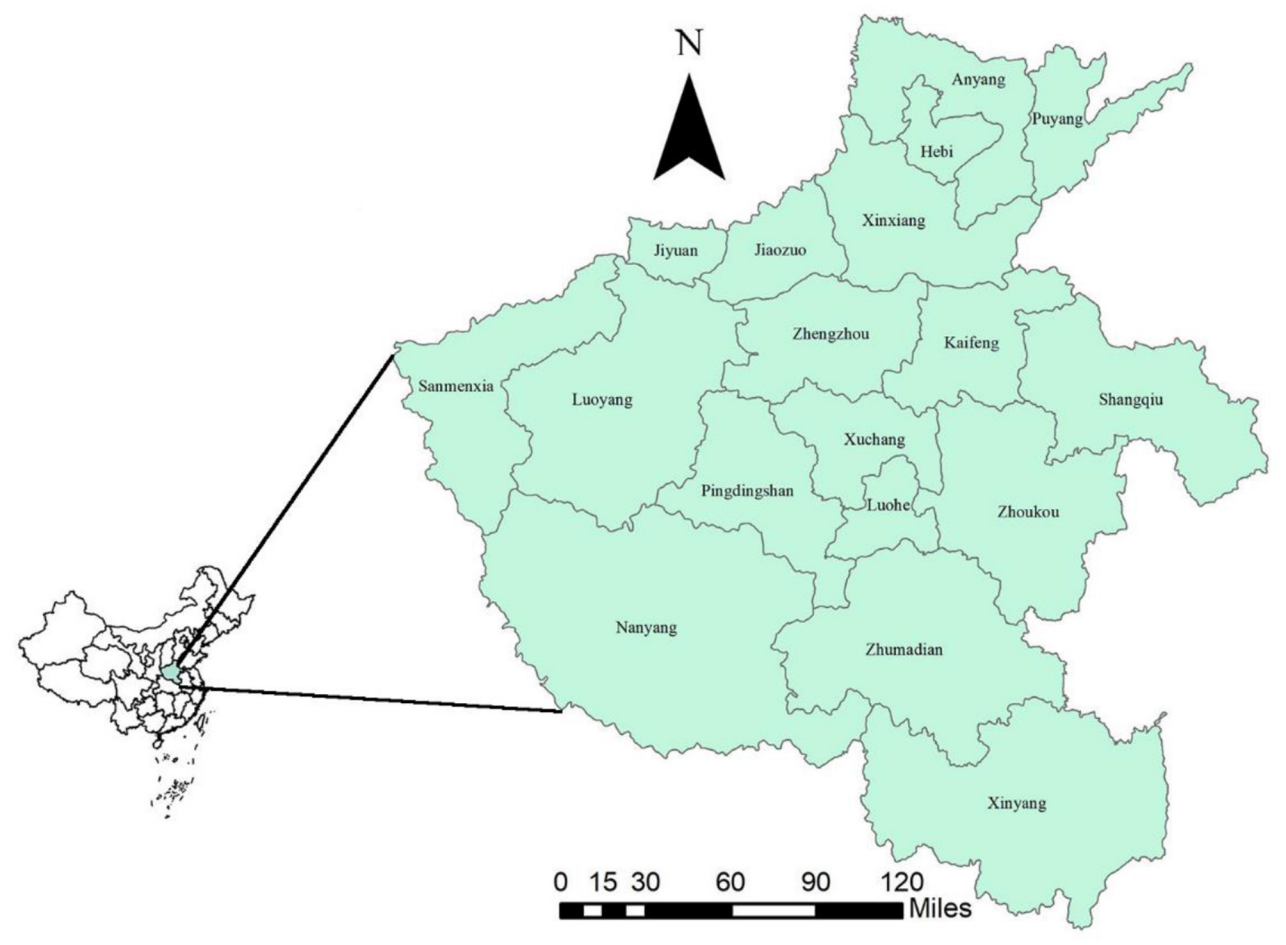
Location of Henan Province (the map was created with ArcGIS software, 10.5).

The study area is located in east-central China (31°23′ to 36°22′ north latitude, 110°21′ to 116°39′ east longitude, Figure 1), with a population of more than 96 million and an area of 167,000 km^2^. Most of Henan is located in the warm temperature zone and has the characteristics of climate transition from plains to hills and mountains from east to west.

### Source of Data

All data were obtained from the official websites of Provincial and Municipal Health Commissions (Supplementary Table 1), which published COVID-19 case data and information. The case data included the number of newly confirmed cases, cured cases, and deaths per day. The case information included age, gender, exposure history, date of symptom onset, and activity trajectory of confirmed cases. Identifiable personal information was removed for privacy protection.

### Case Definition

Although the definition of COVID-19 cases has been changed several times, which has greatly affected the observed epidemic curve in Wuhan (19), the change of cases in Henan Province has been relatively stable, and the diagnosis of all cases in this study were based on the sixth edition of Diagnosis and Treatment Scheme for COVID-19 released by the National Health Commission of China (20). A laboratory-confirmed case was defined if the patient had a positive test of SARS-CoV-2 virus by real-time reverse-transcription-polymerase-chain-reaction (RT-PCR) assay or high-throughput sequencing of nasal and pharyngeal swab specimens. Only laboratory-confirmed cases were included in this study.

### Modeling the Epidemic of COVID-19 in Henan Province

**Figure 2:**
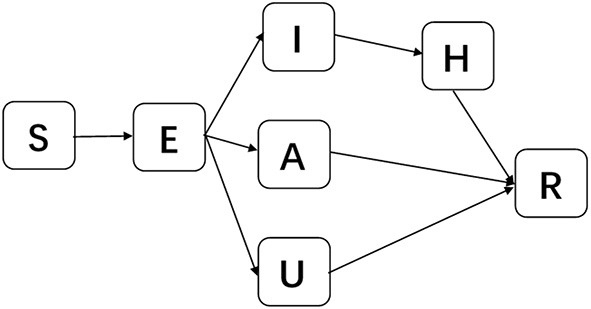
Schematic diagram of SEAIUHR model propagation process. S, susceptible; E, latent; A, asymptomatic infectious; I, confirmed symptomatic infectious; U, unconfirmed symptomatic infectious; H, hospitalized; R, removed.

To consider asymptomatic infected individuals, we constructed the susceptible-exposed-asymptomatic-confirmed-unconfirmed symptomatic-hospitalized-removed (SEAIUHR) model by extending the classic susceptible-exposed-infectious-removed (SEIR) model to include asymptomatic cases, unconfirmed symptomatic cases who did not seek medical attention or get tested for mild symptoms, and quarantined confirmed cases. In this model, we divided the population into seven compartments: S (susceptible), E (latent), A (asymptomatic infectious), I (confirmed symptomatic infectious), U (unconfirmed symptomatic individuals), H (hospitalized), and R (removed). Susceptible individuals could acquire the virus after contact with infected cases (both symptomatic and asymptomatic) and became latent when they were infected but non-infectious. After a period of time, some of the latent individuals developed into symptomatic infections; some of these were confirmed and treated until they progressed into the removed stage and some went unconfirmed because they did not present themselves to healthcare facilities or get tested for mild symptoms. Others developed into asymptomatic infections and remained infectious until they progressed into the removed stage. Removed stage included individuals who were recovered or had died (Figure 2).

Dynamics of these seven parts over time could be expressed by the following ordinary differential equation:

$$\{\begin{array}{ccc} \;\frac{dS}{dt} & = & -\frac{\beta_{t}S(I+U)}{N}-\frac{\beta_{t}\theta SA}{N}\\ \frac{dE}{dt} & = & \frac{\beta_{t}S(I+U)}{N}+\frac{\beta_{t}\theta SA}{N}-\frac{E}{z}\\ \frac{dI}{dt} & = & \frac{\left(1-\mu_{1}-\mu_{2}\right)E}{z}-\frac{I}{r_{1}}\\ \frac{dA}{dt} & = & \frac{\mu_{1}E}{z}-\frac{A}{r_{2}}\\ \frac{dU}{dt} & = & \frac{\mu_{2}E}{z}-\frac{U}{r_{3}}\\ \frac{dH}{dt} & = & \frac{I}{r_{1}}-\frac{H}{r}\\ \frac{dR}{dt} & = & \frac{H}{r}+\frac{A}{r_{2}}+\frac{U}{r_{3}} \end{array}$$

where β_*t*_ was the transmission rate due to symptomatic infected individuals at time *t*, defined as the proportion of cases from susceptible individuals to infected individuals, both asymptomatic and symptomatic, caused by symptomatic infected cases; θ was the ratio of the transmission rate due to asymptomatic over symptomatic cases; μ_1_ and μ_2_ were the proportion of the asymptomatic and unconfirmed symptomatic cases among infected individuals, respectively; *z* was the latent period; *r*_1_, *r*_2_, and *r*_3_ were infectious periods of confirmed symptomatic, asymptomatic, and unconfirmed symptomatic cases, respectively; and *r* was the duration from hospitalization to recovery or death. Assume that *N* = *S* + *E* + *I* + *A* + *U* + *H* + *R*.

The differential equations in the model were numerically solved using a 4th order Runge-Kutta (RK4) method. Specifically, for each step of the algorithm, each term on the right side of the equation was determined using a random sample of the Poisson distribution (5).

On January 25, 2020, Henan Province implemented a first-level public health emergency response to the epidemic and took a series of prevention and control measures, such as traffic restriction, quarantine, contact tracing, isolated treatment of confirmed cases, and so on (21, 22). We assumed that these major government measures caused the transmission rate to change from a constant rate to a time dependent exponentially decreasing rate (23).

Then, the formula of β_*t*_ could be expressed by the following step function:

$$\beta_{t}=\{\begin{array}{c} \;\beta_{0}\;,t<=t_{1}\\ \;\beta_{0}^{*}\exp(-a^{*}(t-t_{1}))\;,t>t_{1} \end{array}$$

where β_0_ was the transmission rate due to symptomatic infected individuals before implementing measures; *a* was the decreasing rate of transmission rate; and *t*_1_ was the date to start implementing measures.

The effective reproductive number, *R*_*t*_, could be computed as:

$$R_{t}=\left(1-\mu_{1}-\mu_{2}\right)\beta_{t}r_{1}+\mu_{1}\theta \beta_{t}r_{2}+\mu_{2}\beta_{t}r_{3}$$

In the initial state, namely, *t* = 0, *R*_*t*_ = *R*_0_ is the basic reproductive number.

### Estimation of Parameters in the Model

Initial states and parameter's setting in the model were presented in Table 1. We assumed that the initial latent population, asymptomatic infected population, and unconfirmed symptomatic cases were drawn from uniform distribution [0,10], the initial confirmed symptomatic infected population was 0, and the rest of Henan Province were susceptible. For parameters, we estimated β_0_, μ_1_, μ_2_, θ, and α by assuming that the values of parameters *z*, *r*_1_, *r*_2_, *r*_3_, and *r* were fixed throughout the process. We assumed that the initial values of each parameter to be estimated were drawn using Latin hypercube sampling in uniform distribution. The initial ranges of μ_1_, μ_2_, and θ were chosen to cover most possible values, i.e. [0,1]; the initial range of α was selected to more broadly cover what the previous research covered (23). The initial range of β was selected from the widest possible range of basic reproductive number (*R*_0_).

**Table 1:** Initial states and parameter's setting in the model of the main analysis.

**States or parameters**	**Values or prior distribution**
**States**	
Susceptible (*S*_0_)	96050000-*E*_0_*-I*_0_*-A*_0_*-U*_0_
Latent (*E*_0_)	*U*(0, 10)
Confirmed symptomatic infectious (*I*_0_)	0
Asymptomatic infectious (*A*_0_)	*U*(0, 10)
Unconfirmed symptomatic infectious (*U*_0_)	*U*(0, 10)
**Parameters**	
Latent period (*z*)	3 days (Fixed) (2, 24–27)
Infectious period of confirmed symptomatic cases (*r*_1_)	3.5 days (Fixed) (5, 22, 28, 29)
Infectious period of asymptomatic cases (*r*_2_)	5 days (Fixed) (17)
Infectious period of unconfirmed symptomatic cases (*r*_3_)	5 days (Fixed) (17)
Duration removed from hospitalization (*r*)	10 days (Fixed) (30)
Transmission rate due to symptomatic cases (β_0_)	*U*(0.8, 1.5) (*Estimated*)
Asymptomatic rate (μ_1_)	*U*(0.02, 1) (*Estimated*)
Undetected rate (μ_2_)	*U*(0.02, 1) (*Estimated*)
The ratio of transmission rate (θ)	*U*(0.02, 1) (*Estimated*)
The decreasing rate of transmission rate (α)	*U*(0.02, 0.3) (*Estimated*)

We used the Ensemble Adjustment Kalman Filter (EAKF) to infer epidemiological parameters of the model based on the number of cases presenting symptoms per day in Henan Province (31–33). The EAKF is a data assimilation algorithm that only needs hundreds of ensembles to obtain good results, especially suitable for the estimation of high-dimensional parameters of the model (34, 35), and has been successfully applied to epidemics such as cholera and influenza (32, 35). In this study, we used 1,000 ensembles and 1,000 independent realizations to infer parameters and their corresponding 95% confidence intervals (Cls).

### Sensitivity Analysis

#### Synthetic Testing

Before applying the model-inference framework to the number of daily incidence data, we tested the effect of model-inference framework with model-generated outbreak data. Specifically, we fixed the parameters of the model to specified values and used the model to generate synthetic outbreak data. We then applied the EAKF algorithm to the synthetic daily outbreak data and assessed the model-inference framework by analyzing whether the model could fit the synthetic outbreak data and estimate parameters.

#### Sensitivity of Parameters Estimation to the Range of Initial States and Values of Fixed Parameters

In initial states, the quantities of *E*_0_, *A*_0_, and *U*_0_ were unknown, and our assumptions may affect the estimation of other parameters. Therefore, this study simultaneously investigated the results of parameter's estimation when shortening and expanding their ranges. At the same time, we changed values of fixed parameters, respectively, to test the robustness of our results.

## Results

**Figure 3:**
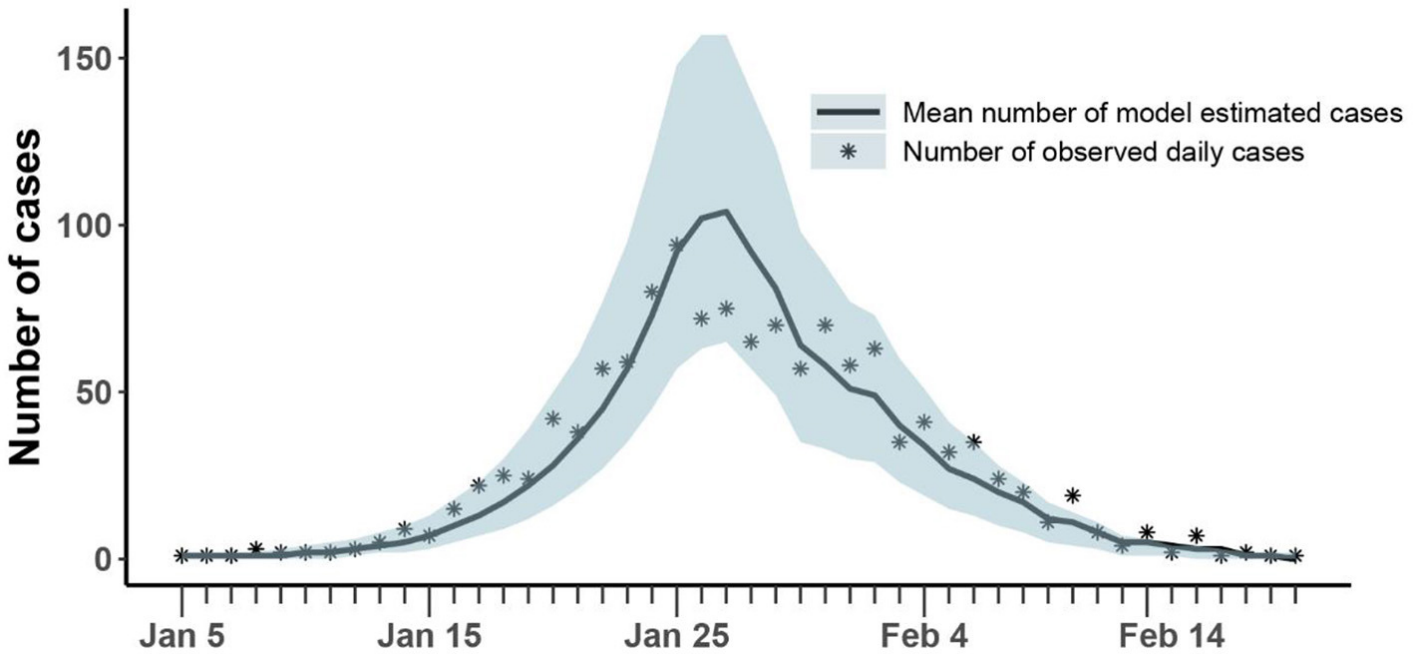
Comparison of the number of cases estimated and observed. The asterisk represents the number of cases with symptoms observed on a daily basis; the curve shows the change in the average number of confirmed symptomatic cases per day estimated by the model. The light blue shade was the 95% confidence interval of the estimation.

As shown in Figure 3, our model could fit reported daily incidence data well and accurately capture the peak and tendency of the epidemic. The numbers of reported daily cases were within the confidence interval estimated by the model, except for a few days in the later stages of the outbreak.

The mean estimation of transmission rate due to symptomatic infected individuals was 1.14 (95% CI:1.07-1.23) at the beginning of the epidemic and the decreasing rate of transmission rate after implementing prevention and control measures was 0.16 (95% CI: 0.12-0.19). Our model estimated that the asymptomatic rate among COVID-19 infected individuals was 42% (95% CI: 41-47%), and the mean ratio of the transmission rate of asymptomatic over symptomatic cases was 0.1 (95% CI: 0.02-0.11). At the same time, our model estimated that 11% (95% CI: 9-22%) of infected individuals were unconfirmed symptomatic cases who did not seek medical attention or get tested for mild symptoms (Table 2). Then, the fraction of undocumented infections in Henan Province was 53% (95% CI: 50-68%). Based on above parameters, we estimated the average effective reproduction number, *R*_*t*_, to be 2.73(95% CI: 2.64-3.31) at the beginning of the epidemic, which was equal to the basic reproduction number (*R*_0_). With the implementation of measures, *R*_*t*_ fell below 1 on January 31.

**Table 2:** Posterior estimates of key epidemiological parameters.

**Parameter**	**Mean (95% CI)**
Transmission rate due to symptomatic cases (β_0_)	1.14 (1.07, 1.23)
Asymptomatic rate (μ_1_)	0.42 (0.41, 0.47)
Undetected rate (μ_2_)	0.11 (0.09, 0.22)
The ratio of transmission rate (θ)	0.10 (0.02, 0.11)
The decreasing rate of transmission rate (α)	0.16 (0.12, 0.19)

*This table showed posterior estimates of key epidemiological parameters using proposed model-inference framework. In the second column, estimated mean and 95% confidence interval were outside and inside parentheses, respectively*.

**Figure 4:**
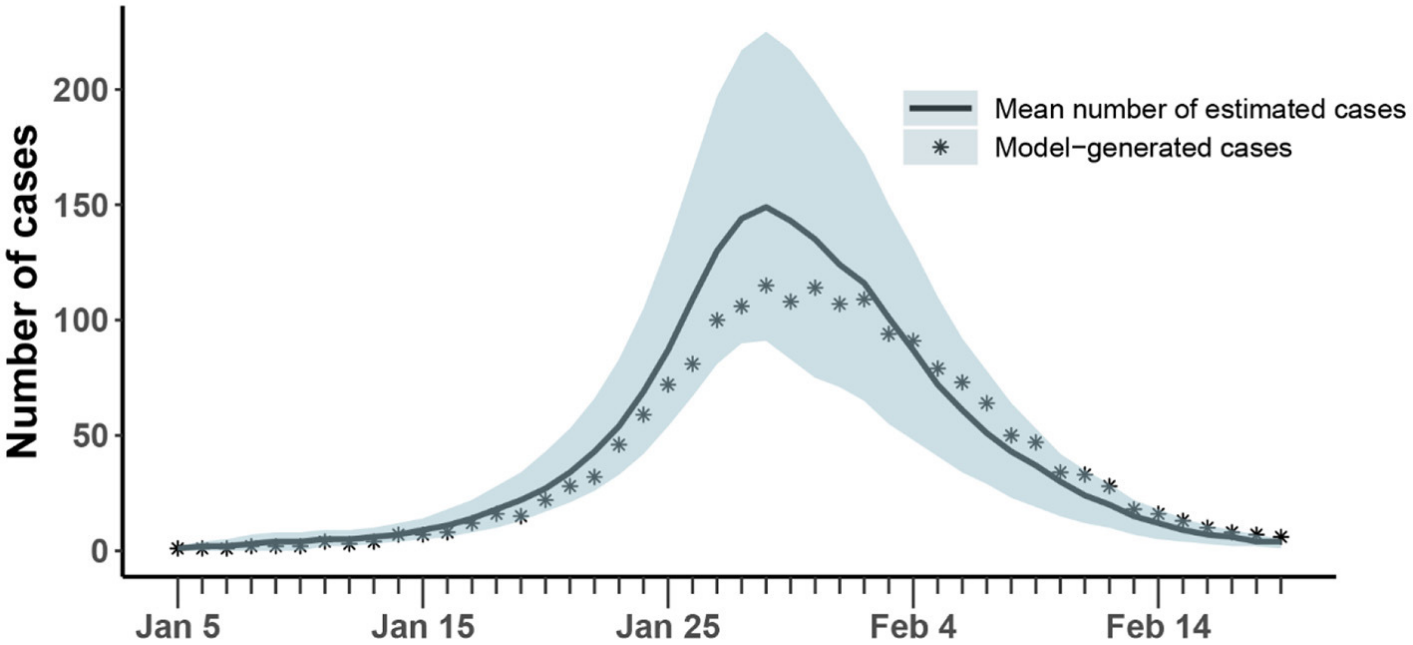
Comparison of the number of cases estimated and generated. The asterisk represents the number of cases with symptoms observed on a daily basis; the curve shows the change in the average number of confirmed symptomatic cases per day estimated by the model. The light blue shade was the 95% confidence interval of the estimation.

The results of the synthetic test were shown in Figure 4 and Table 3. All generated values were within the confidence interval estimated by the model and values of all parameters were within the estimated 95% confidence interval, which demonstrated the ability of the model-inference-framework to fit the synthetic outbreak data and estimate all five target model parameters accurately.

**Table 3:** Results of synthetic testing.

**Parameters**	**True values**	**Estimated values^a^**
β	1.1	1.14 (1.06, 1.22)
μ_1_	0.4	0.34 (0.32, 0.45)
μ_2_	0.1	0.09 (0.07, 0.23)
θ	0.2	0.18 (0.14, 0.22)
α	0.15	0.15 (0.12, 0.19)

a *The estimated mean and 95% confidence interval were outside and inside the parentheses*.

Results of parameter estimation when changing the range of initial states and values of fixed parameters were shown in Supplementary Table 2. It could be seen that values of the resampled epidemiological parameters fall near the values estimated from the original data, with small fluctuations, indicating that the estimated results of our model are robust.

## Discussion

Taking Henan Province as an example, we constructed a SEAIUHR model to estimate the prevalence of asymptomatic COVID-19 cases and their contribution in transmission with EAKF algorithm. This model-inference framework is also applicable to studies of asymptomatic infected individuals in other regions.

Asymptomatic proportion, which is broadly defined as the proportion of asymptomatic infections among all infections of the disease, is important for estimating the true burden of disease and its transmission potential. At present, results of different studies on the asymptomatic proportion vary greatly (15). We estimated that the proportion of asymptomatic infections among infected individuals during the entire epidemic was 42% in Henan Province, within the confidence interval of the estimated asymptomatic rate of 13 cases imported from Wuhan to Japan (14). But it was higher than that of the Diamond Princess cruise ship, which showed that only 17.9% of those infected were asymptomatic (36). It could be that passengers and crew on the Diamond Princess were not drawn from a random sample of the general population, most of whom were older than 60 years and tended to have more severe symptoms after infection. Our model estimated that the mean ratio of transmission rate due to asymptomatic over symptomatic cases was 0.1, corresponding to a study showing that prolonged exposure to infected persons and short exposure to symptomatic persons (such as coughing) is associated with a higher risk of transmission, while short exposure to asymptomatic contacts is associated with a lower risk of transmission (24). The less contagious nature of asymptomatic individuals may be the result of a convolution of the shedding fraction of viable virus, the titer of viable virus in the primary/upstream case, and possibly behavioral factors.

The fraction of undocumented infections, including asymptomatic cases and unconfirmed symptomatic cases who did not seek medical attention or get tested for mild symptoms, was lower than that of Wuhan in the early stage of the epidemic (5, 6, 8), which may be caused by following reasons. Firstly, in the early stage, the medical configuration was not perfect and public awareness was still insufficient, while the undocumented rate gradually decreased with the development of the epidemic (5, 10, 37). Secondly, contact tracing measures implemented in China may become unfeasible when the number of cases in Wuhan rose sharply in the early stage (3). Finally, we need to point out that the differences in the estimated proportions of asymptomatic cases and unconfirmed symptomatic cases may be due to unidentifiability of parameters in epidemiological models. The theoretical analysis of identifiability of parameters in epidemiological models needs to be done in the future.

Basic reproductive number (*R*_0_) is an important parameter to determine whether an infectious disease is prevalent or not. If *R*_0_ < 1, infectious disease would gradually decline and die out without an epidemic; if *R*_0_ > 1, an epidemic would break out. In this study, our estimation of *R*_*t*_ = 2.73 at the beginning of the epidemic measured the basic reproductive number *R*_0_, that is, without intervention, each infected individual could infect an average of 2.73 susceptible individuals. This result was similar to some studies in other regions of China (28, 38, 39), although it was smaller than results from some other research (38). Combined with the latent period, the number of cases without intervention would increase exponentially (25, 29). However, Henan Province implemented a first level response on January 25, 2020, and adopted a series of prevention and control measures. The isolation treatment of confirmed cases and the testing of suspected cases aimed at removing infected individuals from the process of transmission. The closing of public places and the change of crowd behavior were to protect susceptible groups. Contact tracing, which identified possible chains of transmission between known infected persons and their close contacts, affected both susceptible and asymptomatic individuals and can effectively interrupt transmission. With the help of these measures, *R*_*t*_ dropped below 1 on January 31, 2020.

This study also has some limitations. Firstly, our estimation of the asymptomatic proportion and infectivity was obtained by a model, which could not be generalized because it has not been confirmed by serological investigation. Secondly, we only used data from Henan Province, which might limit the interpretation of our results, although our model-inference framework is also applicable to studies of asymptomatic infected individuals in other regions. Therefore, large-scale relevant studies are needed in the future. Thirdly, this study estimated the average asymptomatic infection rate in Henan Province over time, but the asymptomatic rate may vary in different periods of the epidemic.

## Conclusion

The epidemic situation developed rapidly in Henan Province, and there were a large number of asymptomatic infected individuals with relatively low infectivity. Our study further explored the prevalence of asymptomatic COVID-19 cases and their contribution to transmission so as to deepen people's understanding of asymptomatic cases and provide a reference for the prevention and control of COVID-19.

## Data Availability Statement

The raw data supporting the conclusions of this article will be made available by the authors, without undue reservation.

## Author Contributions

CL and XL conceived of and designed the research. CL, YZ, CQ, LL, DZ, XW, KS, YJ, and TL did the analyses. CL wrote and revised the paper. DH, MX, and XL contributed to the writing and revisions. All the authors have read and approved the submitted version. All the authors have agreed both to be personally accountable for the author's own contributions and to ensure that questions related to the accuracy or integrity of any part of the work are answered.

## Conflict of Interest

The authors declare that the research was conducted in the absence of any commercial or financial relationships that could be construed as a potential conflict of interest.

## Abbreviations

COVID-19: coronavirus disease 2019
*R*_0_: the reproductive number
*R*_*t*_: the effective reproductive number
SEIAUHR model: susceptible-exposed-asymptomatic-confirmed-unconfirmed symptomatic-hospitalized-removed model.
