# Supplementary material for: Estimating the Prevalence of Asymptomatic COVID-19 Cases and Their Contribution in Transmission - Using Henan Province, China, as an Example
Source: Front Med (Lausanne). 2021 Jun 23;8:591372. doi: 10.3389/fmed.2021.591372 (PMC8260942; doi:10.3389/fmed.2021.591372)
Supplement: Supplementary file 1 [file Table_1.doc]

**Table S1. Source of data**

| Area | Website |
| --- | --- |
| Henan Province | http://wsjkw.henan.gov.cn/ |
| Zhengzhou Shi | http://wjw.zhengzhou.gov.cn/ |
| Kaifeng Shi | http://www.kfwsjsw.gov.cn/ |
| Luoyang Shi | http://www.lyws.gov.cn/ |
| Pingdingshan Shi | http://www.pdswsjsw.gov.cn/ |
| Anyang Shi | http://aywjw.anyang.gov.cn/ |
| Hebi Shi | https://wsjkw.hebi.gov.cn/ |
| Xinxiang Shi | http://www.xxswjw.gov.cn/ |
| Jiaozuo Shi | http://www.jzswjw.gov.cn/ |
| Puyang Shi | http://www.pyswjw.gov.cn/ |
| Xuchang Shi | http://xcswjw.xuchang.gov.cn/ |
| Luohe Shi | http://www.lhswjw.gov.cn/ |
| Sanmenxia Shi | <http://wjw.smx.gov.cn/> |
| Nanyang Shi | http://nyws.nanyang.gov.cn/ |
| Shangqiu Shi | http://www.sqwsjd.cn/ |
| Xinyang Shi | http://www.hnxywjw.gov.cn/ |
| Jiyuan Shi | http://www.zkwjw.gov.cn/ |
| Zhumadian Shi | http://www.zmdwsj.gov.cn/ |
| Jiyuan Shi | http://wjw.jiyuan.gov.cn/ |

**Table S2. Results of parameters estimation when values of initial states or fixed parameters changed**

|  | 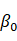 | 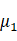 | 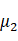 | 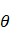 | 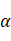 |
| --- | --- | --- | --- | --- | --- |
| C1^a^ | 1.14(1.05,1.22) | 0.44(0.42,0.47) | 0.09(0.07,0.21) | 0.08(0.02,0.09) | 0.15(0.12,0.18) |
| C2^b^ | 1.13(1.06,1.22) | 0.43(0.42,0.47) | 0.16(0.15,0.4) | 0.09(0.02,0.10) | 0.16(0.12,0.19) |
| C3^c^ | 1.14(1.06,1.22) | 0.43(0.43,0.47) | 0.16(0.15,0.39) | 0.09(0.02,0.10) | 0.16(0.12,0.19) |
| 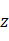=2^d^ | 1.14(1.07,1.23) | 0.45(0.44,0.47) | 0.15(0.10,0.21) | 0.08(0.02,0.09) | 0.15(0.12,0.18) |
| 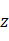=4^e^ | 1.15(1.07,1.23) | 0.39(0.38,0.46) | 0.07(0.05,0.25) | 0.09(0.03,0.11) | 0.15(0.12,0.19) |
| 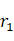=2^f^ | 1.14(1.06,1.22) | 0.37(0.36,0.46) | 0.12(0.10,0.29) | 0.14(0.08,0.16) | 0.15(0.12,0.18) |
| 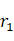=3^g^ | 1.13(1.06,1.22) | 0.42(0.41,0.47) | 0.15(0.14,0.33) | 0.10(0.05,0.12) | 0.16(0.13,0.19) |
| 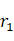=4^h^ | 1.14(1.06,1.22) | 0.44(0.43,0.47) | 0.12(0.10,0.28) | 0.08(0.02,0.08) | 0.16(0.12,0.19) |
| 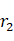=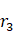=4^i^ | 1.14(1.06,1.22) | 0.37(0.35,0.46) | 0.16(0.14,0.32) | 0.18(0.14,0.22) | 0.15(0.12,0.19) |
| 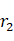=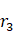=6^j^ | 1.14(1.07,1.23) | 0.40(0.39,0.47) | 0.17(0.15,0.34) | 0.12(0.07,0.15) | 0.16(0.12,0.19) |
| 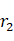=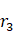=7^k^ | 1.14(1.06,1.22) | 0.40(0.39,0.47) | 0.14(0.13,0.37) | 0.11(0.03,0.12) | 0.16(0.12,0.19) |

The table showed the estimated results of each parameter when the initial range of certain states or fixed parameters changed, where the estimated mean value was outside the parenthesis and the 95% confidence interval was inside the parenthesis.

^a^change the initial range of
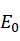
,
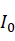
, and
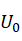
 to [0,5].

^b^change the initial range of
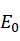
,
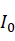
, and
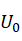
 to [0,15].

^c^change the initial range of
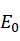
,
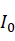
, and
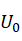
 to [0,20].

^d^Change the value of
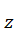
 to
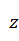
=2.

^e^Change the value of
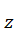
 to
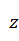
=4.

^f^Change the value of
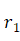
 to
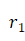
=2.

^g^Change the value of
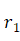
 to
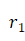
=3.

^h^Change the value of
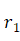
 to
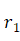
=4.

^i^Change the value of
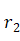
 and
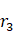
 to
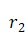
=
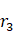
=4.

^j^Change the value of
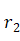
 and
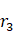
 to
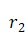
=
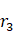
=6.

^k^Change the value of
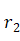
 and
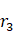
 to
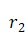
=
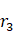
=7.
